# Electrospun PCL Scaffolds as Drug Carrier for Corneal Wound Dressing Using Layer-by-Layer Coating of Hyaluronic Acid and Heparin

**DOI:** 10.3390/ijms23052765

**Published:** 2022-03-02

**Authors:** Marcus Himmler, Dirk W. Schubert, Lars Dähne, Gabriella Egri, Thomas A. Fuchsluger

**Affiliations:** 1 Department of Ophthalmology, University Medical Center Rostock, Doberaner Straße 140, 18057 Rostock, Germany; 2 Institute of Polymer Materials, Friedrich-Alexander University Erlangen-Nuremberg, Martenstraße 7, 91058 Erlangen, Germany; dirk.schubert@fau.de; 3 Surflay Nanotec GmbH, Max-Planck-Str. 3, 12489 Berlin, Germany; l.daehne@surflay.com (L.D.); g.egri@surflay.com (G.E.)

**Keywords:** electrospinning, polycaprolactone, cornea, wound dressing, layer-by-layer coating, hyaluronic acid, heparin, suture retention test, drug delivery

## Abstract

Due to its ability to reduce scarring and inflammation, human amniotic membrane is a widely used graft for wound dressings after corneal surgery. To overcome donor dependency and biological variances in the donor tissue, artificial nanofibrous grafts acting as drug carrier systems are promising substitutes. Electrospun nanofibrous scaffolds seem to be an appropriate approach as they offer the properties of permeable scaffolds with a high specific surface, the latter one depending on the fiber diameter. Electrospun scaffolds with fiber diameter of 35 nm, 113 nm, 167 nm and 549 nm were manufactured and coated by the layer-by-layer (LbL) technology with either hyaluronic acid or heparin for enhanced regeneration of corneal tissue after surgery. Studies on drug loading capacity and release kinetics defined a lower limit for nanofibrous scaffolds for effective drug loading. Additionally, scaffold characteristics and resulting mechanical properties from the application-oriented characterization of suture pullout from suture retention tests were examined. Finally, scaffolds consisting of nanofibers with a mean fiber diameter of 113 nm were identified as the best-performing scaffolds, concerning drug loading efficiency and resistance against suture pullout.

## 1. Introduction

The current gold standard for wound dressings after corneal surgery is the use of human amniotic membranes (hAM) and female placenta tissue. Their beneficial properties are well-known, such as enhancing re-epithelization or anti-inflammatory and antimicrobial activities [1]. Usually, the tissue is sutured to the patient’s eye due to persistent epithelial defects or corneal ulceration [2]. As with most allogenic tissues for the use in tissue engineering, concerns due to variations in quality, donor shortage or potential pathogens exist [3]. To overcome these disadvantages, research is focused on artificial wound dressings [4,5]. Electrospun nanofibrous scaffolds offer the possibility to produce highly permeable scaffolds with adjustable properties, like fiber diameter, scaffold thickness, biodegradability and biocompatibility. Especially in the field of corneal surgery, the permeability of nanofibrous scaffolds, allowing the naturally occurring exchange of metabolic products and nutrition for the epithelial cells, are of major importance. Moreover, in comparison to conventional films, nanofibrous scaffolds inherit a huge specific surface, offering the possibility for specific surface functionalization, for example absorption of anti-inflammatory and antimicrobial agents or growth factors to enhance re-epithelization. In the literature, various approaches towards the functionalization of nanofibrous scaffolds were discussed, e.g., in [6], in which the layer-by-layer (LbL) coating represents a feasible technique [7,8]. Chitosan-based LbL-coating is a well-known approach due to the polycationic nature of chitosan in acidic conditions [9]. Furthermore, chitosan-based LbL-coating offers several features, based on the beneficial properties of chitosan, e.g., antimicrobial activity [10,11,12]. Thus, the chitosan-based LbL-coating offers a direct method for the absorption of molecules, for example desired drugs, to the nanofibers, thus expanding the beneficial properties of artificial wound dressings. Using the LbL technology, the amount as well as the release of the drug can be tuned and scaffolds with distinctive properties, according to the requirements of the later application, can be produced. In contrast to the LbL-coating of nanoparticles as drug carrier (e.g., [13]), our approach was to directly immobilize the drugs on the nanofiber surface.

In the present study, polycaprolcatone (PCL) was used for the fabrication of nanofibrous scaffolds. Its good miscibility with natural polymers and biodegradability as well as cytocompatibility led to the extended use of this polymer in the field of tissue engineering, especially corneal tissue engineering [14]. Postsurgery, several drugs are reasonable for aftercare like hyaluronic acid or heparin. Hyaluronic acid, a polysaccharide, is known for its inflammatory effects, fostering cell proliferation and wound healing [15,16,17,18]. Moreover, the hygroscopic nature of the polysaccharide ensures for a high water-storing capacity [19]. Previous studies have already shown the advantageous properties of polysaccharide LbL-coated nanofibers in preliminary cell culture experiments [20]. In addition to hyaluronic acid, LbL-coating of heparin was performed. Heparin is well-known to prevent bloodstream infections and its anticoagulation properties, enhancing long-term patency of heparin coated grafts in the body [12,21]. Therefore, in this study, the influence of fiber diameter on drug loading and release kinetics is investigated and mechanical properties in the application-oriented suture retention test (SRT) were evaluated to further develop functionalized nanofibrous scaffolds for corneal wound healing. Therefore, in a first step, the nanofibrous scaffolds were characterized regarding areal weight, scaffold density and porosity. Based on these findings, results from mechanical tests could be normalized, thus scaffolds consisting of nanofibers with different fiber diameter could be compared. The drug release from the scaffolds was investigated and resulting drug loading capacity evaluated and finally, the overall scaffold performance regarding resistance against suture pullout and drug loading could be determined.

## 2. Results and Discussion

### 2.1. Scaffold Characterisitcs

Properties of electrospun scaffolds are mainly defined by the material, fiber diameter and spinning time. Resulting characteristics are scaffold thickness, porosity and consequently areal weight and scaffold density.

**Figure 1:**
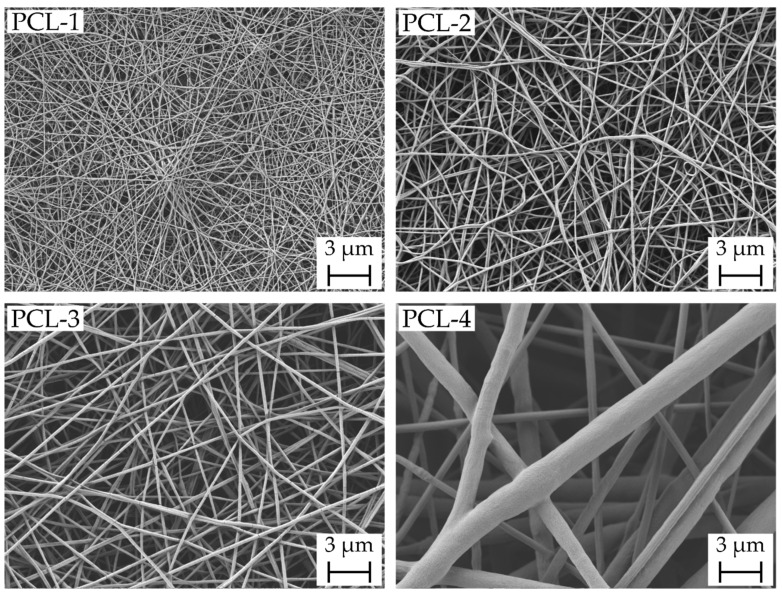
With increasing solution concentration and varying solvent system polycaprolactone (PCL) nanofibrous scaffolds consisting of nanofibers with a mean fiber diameter of 35 ± 13 nm (PCL-1), 113 ± 22 nm (PCL-2), 167 ± 35 nm (PCL-3) and 549 ± 225 nm (PCL-4) were produced.

The resulting scaffolds are shown in Figure 1 in the scanning electron microscopy (SEM) images with a magnification of 10,000. As reported in Table 1, the mean fiber diameter increases from 35 nm (PCL-1) to 549 nm (PCL-4). Within this range of fiber diameter, differences in scaffold characteristics, resistance against suture pullout and drug carrying properties were investigated. The broad fiber distribution for the PCL-4 scaffolds presumably originates from the solvent system chloroform/ethanol and corresponding high evaporation rates of the solvent system enhancing fiber splitting according to Schubert [22,23].

**Figure 2:**
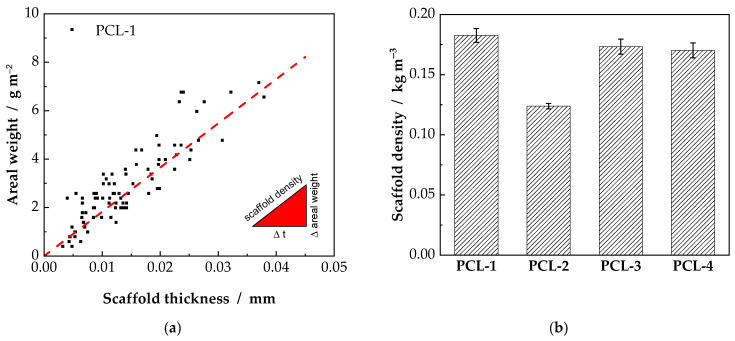
Exemplary chart for the PCL-1 scaffold (**a**). Scaffold density can be calculated as the slope of the areal weight vs. scaffold thickness, as depicted in Equation (5). The resulting densities are displayed as bar chart in (**b**).

As depicted in Equation (5), the scaffold density can be calculated as the slope of the areal weight versus scaffold thickness. An example for the PCL-1 samples is given in Figure 2a and calculated scaffold densities are shown in Figure 2b. Within the experimental error, no significant difference of the scaffold densities of the PCL-1, PCL-3 and PCL-4 scaffolds can be observed. For these samples, a scaffold density of 0.17 to 0.18 kg m^−3^ was measured. It has to be pointed out that the PCL-2 scaffolds differ significantly from the other samples with a scaffold density of 0.12 kg m^−3^. In comparison to the PCL-1 and PCL-3 scaffolds, which were produced from the same solvent system, only the needle-to-collector distance in the electrospinning process was altered (the slightly different flow rates are to be neglected). The increased time for the polymer jet to solidify before reaching the collector results in stiffer fibers. These fibers are consequently less likely to be collected in a compact manner, reducing the scaffold density. The values for the scaffold densities indicate that the scaffold density is not only influenced by the fiber diameter, but also by the electrospinning process, in this case namely the needle-to-collector distance.

From the scaffold densities, the porosity of the fabricated scaffolds can be calculated, as depicted in Equation (6). Scaffold densities and resulting scaffold porosities are summarized in Table 1. For all samples, resulting porosity values indicate a very low packing density of the fibers. With the exception of the PCL-2 scaffolds, a porosity of approximately 85% can be observed. In contrast, the PCL-2 scaffold possesses a porosity of 89%, as already indicated by the scaffold density.

### 2.2. Suture Retention Test

**Figure 3:**
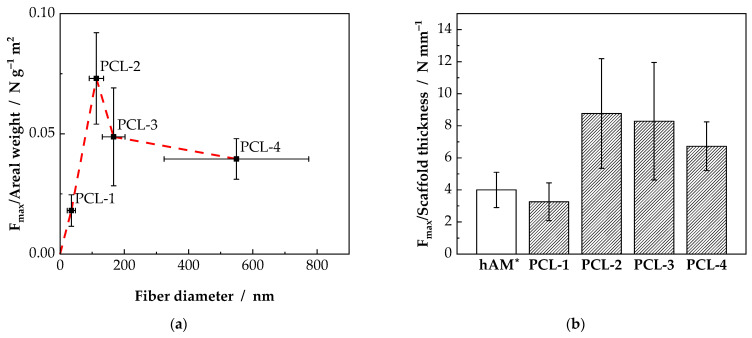
Results of the suture retention test for scaffolds consisting of fiber with a diameter of 35 nm (PCL-1), 113 nm (PCL-2), 167 nm (PCL-3) and 589 nm (PCL-4). (**a**) The resistance against suture pullout is displayed as the maximum force normalized to the areal weight of the samples. (**b**) Comparison of the polycaprolactone (PCL) scaffolds with human amniotic membrane (hAM) concerning the force normalized to the scaffold thickness. * Adapted with permission from Ref. [24]. Copyright 2022 Elsevier.

The suturability of the scaffolds is crucial for the later application as wound dressing. The characteristic parameters are the maximum force the scaffold can withstand as well as the deformation at F_max_ and the deformation at suture pullout, as depicted in Figure 8. In Figure 3a, the mean maximum force normalized to the areal weight of the sample is shown. The lowest value is observed for the PCL-1 samples, consisting of nanofibers with a fiber diameter of 35 nm, with 0.018 N g^−1^ m^2^. The PCL-2 and PCL-3 scaffolds show a significantly increased F_norm_, whereby the highest value is observed for the PCL-2 samples with 0.073 N g^−1^ m^2^. Even though the PCL-4 samples have the largest fiber diameter, the normalized maximum force is rather small with 0.040 N g^−1^ m^2^. The results indicate that increasing fiber diameter does not necessarily increase suture retention. Furthermore, based on the results from Section 2.1, it can be concluded, that a reduced areal weight does not reduce the suture resistance the same way. The scattering of the measurements is high with relative errors of 21% (PCL-2) to 42% (PCL-3).

The trend, indicated by the dashed line in Figure 3a, could be explained as superposition of two opposite effects. On the one hand, with decreasing fiber diameter, the scaffolds’ homogeneity is increasing and thus reducing imperfections, resulting in an improved resistance against suture pullout. With decreasing fiber diameter from PCL-4 to PCL-2 the resistance against suture pullout is increasing from 0.040 N g^−1^ m^2^ to 0.073 N g^−1^ m^2^. On the other hand, the mechanical properties of nonwovens is highly dependent on the fiber-fiber connections. With decreasing fiber diameter, the specific fiber surface is increasing and thus evaporation of the solvents during the electrospinning process. Consequently, upon fiber collection, the nanofibers are less likely to build stable nodes, resulting in a significantly reduced resistance against suture pullout. Accordingly, for the scaffolds in this study, a sweet spot can be identified for the PCL-2 scaffolds.

To compare the resulting data with the human amniotic membrane (hAM) as benchmark material, F_norm_ was recalculated as force normalized to the scaffold thickness in N mm^−1^. Combining Equations (5) and (7) the force normalized to the scaffold thickness can be calculated using the scaffolds density ρ_scaffold_. For the tested samples, as shown in Figure 3b, values of 3.26 N mm^−1^ (PCL-1), 8.77 N mm^−1^ (PCL-2), 8.29 N mm^−1^ (PCL-3) and 6.73 N mm^−1^ (PCL-4) were calculated. Thus, for the PCL-2, PCL-3 and PCL-4 samples, an increased resistance against suture pullout compared to hAMs (4.0 N mm^−1^ [24]) was measured. Subsequently, wound dressings could be produced from thinner scaffolds than comparable hAM leading to an enhanced transparency of the scaffolds [25]. The PCL-1 samples reach a resistance against suture pullout comparable to hAMs.

**Figure 4:**
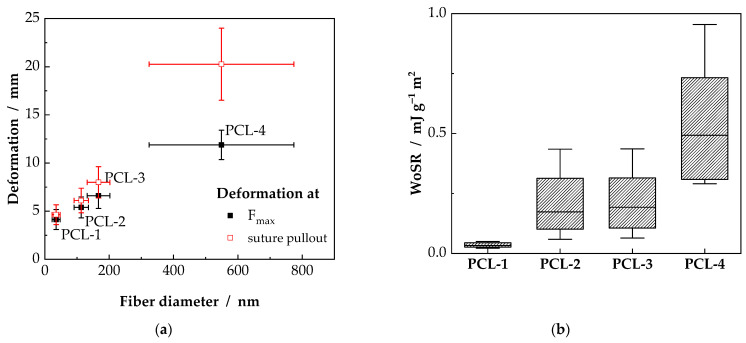
(**a**) Scaffold deformation is shown at F_max_ and suture pullout. (**b**)Whisker box plots of the work of suture retention (WoSR), representing the security against suture pullout. The box indicates the standard deviation and the whiskers the 5% and the 95% percentiles.

Figure 4a shows the deformation at F_max_ as well as the deformation at suture pullout. In this case, an increase in deformation, d_s_ as well as d_r_, can be observed. For the PCL-1 scaffolds only 4.1 mm and 4.6 mm deformation for d_s_ and d_r_ were measured. With increasing fiber diameter, the deformation d_s_ and d_r_ increase to 11.9 mm and 22.3 mm, respectively. Even though, the normalized force of the PCL-4 scaffold is smaller, compared to the PCL-2 and PCL-3 scaffolds, the deformation until suture pullout increases with increasing fiber diameter.

It is noteworthy that the ratio between the deformation at F_max_ and the deformation at suture pullout decreases with increasing fiber diameter. For the samples PCL-1, PCL-2 and PCL-3 d_s_ is at 89%, 88% and 83% of d_r_. For the PCL-4 samples, the maximum force is already reached at 59% of d_r_ and further resistance against suture pullout after reaching F_max_ results in ongoing deformation of the scaffold until failure. The increase in deformation results in a significantly increased work of suture retention (WoSR), as shown in Figure 4b. A clear dependency towards a higher WoSR with increasing fiber diameter can be observed. For the PCL-1 samples a WoSR of (0.04 ± 0.01) 10^−3^ J g^−1^ m^2^ is measured, increasing for the PCL-2 and PCL-3 samples to (0.21 ± 0.10) 10^−3^ J g^−1^ m^2^. The highest WoSR was detected for the samples with the highest fiber diameter with (0.52 ± 0.21) 10^−3^ J g^−1^ m^2^. Thus, the highest work for suture retention is necessary for the PCL-4 samples, adding additional security to the seam.

### 2.3. Drug Release Properties

**Figure 5:**
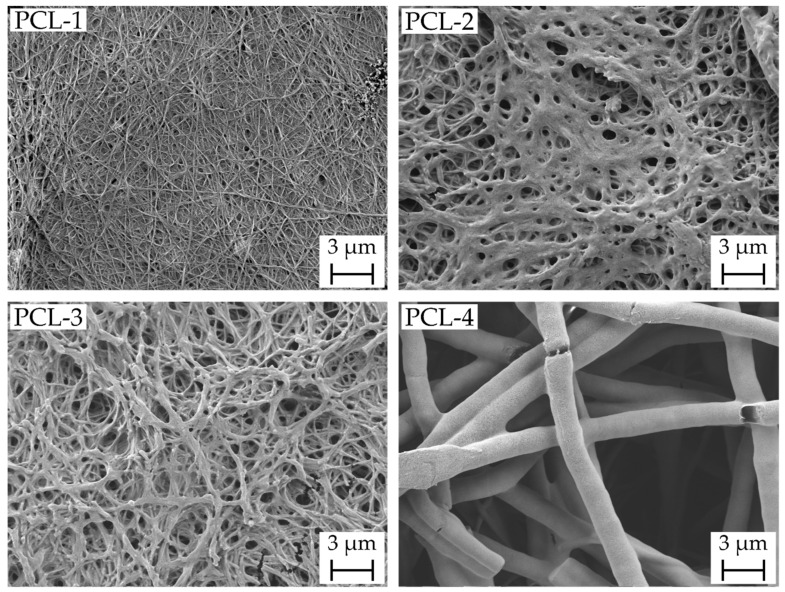
Scanning electron microscopy (SEM) images of the layer-by-layer (LbL)-coated polycaprolactone (PCL) scaffolds. For the PCL-1 scaffolds, the LbL-coating forms an almost dense layer on top of the nanofibrous scaffold. With increasing fiber diameter, the fibrous structure remains intact and coating takes place on the fiber surface.

The influence of fiber diameter on the drug carrier properties of PCL nanofibrous scaffolds is of interest, as it defines the drug loading capacity as well as the release kinetics. In Figure 5, SEM images of the scaffolds after the LbL-coating are shown.

In comparison to the as-spun fibers in Figure 1, the coated layer is clearly visible. Interestingly, the coating almost completely covers the fibrous structure for the PCL-1 samples, thus minimize the accessible surface for the LbL-coating. In addition, exemplary Confocal Laser Scanning Microscopy (CLSM) images of the PCL-2 scaffolds are shown in Figure S4. In theory, the specific surface of a scaffold S_scaffold_, which is available for the absorption of polyanions and polycations in the LbL-coating process, is increasing with decreasing fiber diameter. Assuming an endless fiber, the resulting surface from a defined amount of polymer can be calculated using the formulation for the surface of a cylinder S_surface_ with radius r and length L (neglecting the base area)

(1) $$S_{\mathrm{cylinder}}=2\pi r\;L$$

and the cylinder volume V_cylinder_

(2) $$V_{\mathrm{cylinder}}{=\pi r}^{2}\;L$$

with the density ρ=mV of the material, the mass of the scaffold m and fiber diameter D, Equations (1) and (2) can be solved to

(3) $$S_{\mathrm{scaffold}}=\frac{4m}{\rho D}$$

describing the overall surface of a scaffold of mass m consisting of nanofibers with fiber diameter D. From Equation (3) it becomes clear, that with decreasing fiber diameter the specific scaffold surface increases with S_scaffold_ ~ D^−1^.

**Figure 6:**
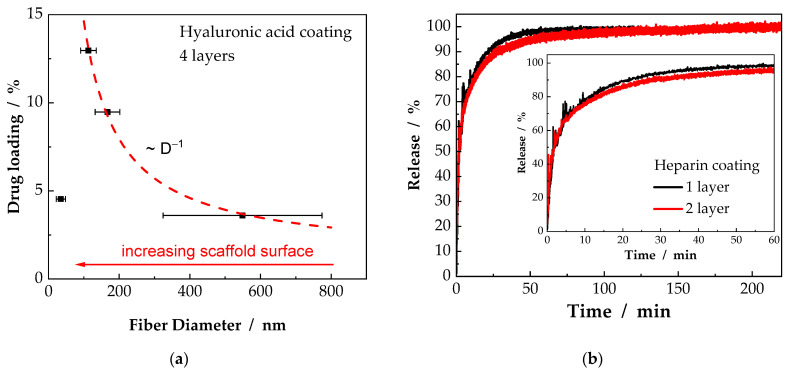
Percentage of absorbed drug per gram scaffold, indicating an increased drug loading capacity with decreasing fiber diameter, whereby a lower limit can be defined at which the potential surface is not fully exploited (**a**). Increasing the number of coated layers results in a decelerated drug release (**b**).

In Figure 6a, the drug loading capacity, as defined in Equation (10), is shown. In addition, the dashed line indicates the specific surface of the scaffolds, dependent on the inverse of the fiber diameter D^−1^. Interestingly, this dependency does not hold for the scaffolds consisting of fibers with a diameter of 35 nm. Even though, these scaffolds should possess the largest surface per gram scaffold, the amount of immobilized drug is significantly lower than to be expected. For the LbL-coating technique, the fiber surface has to be activated with ozone and subsequent polycations and polyanions are adsorbed, as described in Section 3.4. Obviously, the accessibility of the fiber surface is drastically hindered, resulting in insufficient drug immobilization, as indicated by the SEM images in Figure 5. The dense layer inhibits a further coating of the central layers of nanofibers within the scaffolds, resulting in a significantly reduced drug loading capacity. In addition, this dense, superficial layer significantly reduces the desired permeability through electrospun scaffolds, which is usually possible due to the porous structure. Thus, the transfer of metabolic products through the scaffolds is hindered.

Finally, the influence of the number of coated layers is investigated. Therefore, scaffolds with a fiber diameter of 113 nm (PCL-2) were coated. The coating of the nanofibers with one and two layers of heparin, as displayed in Figure 6b, shows the decelerated release with increasing number of coated layers. Meanwhile for both samples t_50%_ is reached after roughly two minutes, the difference becomes more pronounced at t_90%_. For a coating of one layer, 90% of drug is released after 22 min. With the coating of a second layer, the time is extended to 29 min. With only one additional layer it could be shown that the initial release can be inhibited and the drug release is prolonged by over 30%. The unintentional initial release can be attributed to the superficial layers of LbL-coating, which remains constant, even though additional layers will be added. However, while adding more LbL-coated layers, the percentage release decreases as the overall amount of immobilized drug increases. Thus, the initially high release, as indicated in Figure 6b becomes less pronounced and a drug release over a long time can be achieved.

**Figure 7:**
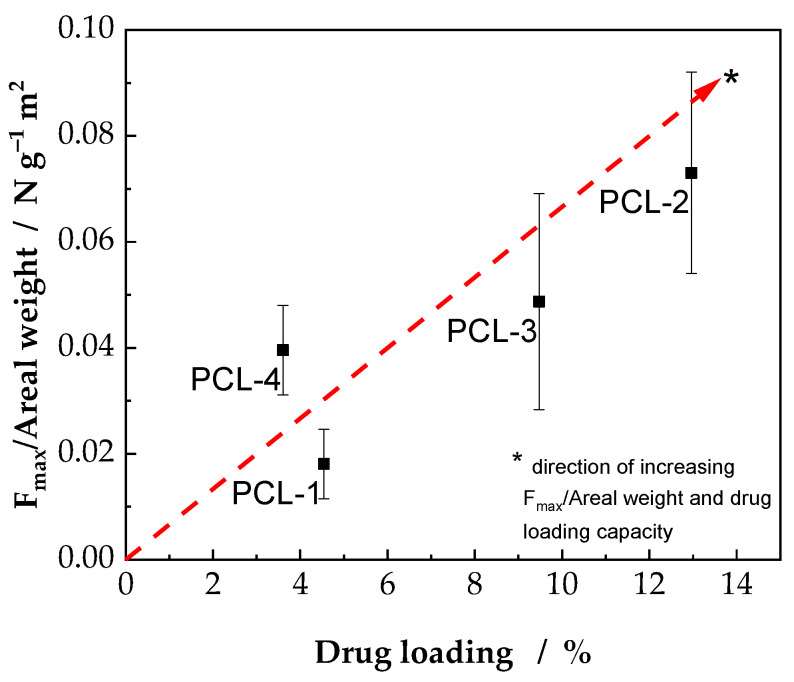
Comparison of resistance against suture pullout and drug loading capacity. As displayed, the PCL-2 scaffold shows the highest resistance against suture pullout and simultaneously high drug loading capacity. The dashed line indicates the direction of increasing suture retention and drug loading capacity.

Combining the results from the suture retention test and drug release studies, a comparison of all four types of scaffolds can be conducted. Therefore, in Figure 7 the resistance against suture pullout was plotted against the drug loading capacity. As indicated previously, the PCL-2 scaffold shows the best result regarding drug loading capacity as well as resistance against suture pullout. Even though the use of the individual components is common practice, the overall system must be examined for its biological applicability in further studies. In addition, the number of LbL-coated layers, corresponding amount of immobilized drug, and their effect on corneal tissue addressing cytotoxic aspects should be addressed. In the present study, a lower fiber diameter limit for an efficient immobilization of drug on electrospun scaffolds using the LbL-coating technology is defined. Meanwhile published literature deals with nanofibers in the range from 350 nm to 1200 nm [20,21], drug immobilization could be significantly enhanced by decreasing the fiber diameter. To our knowledge, so far, no studies were performed on nanofibers with a mean fiber diameter significantly below 100 nm. We could show that a lower limit exists below which the LbL-coating forms a dense barrier on top of the scaffolds, instead of coating the individual fiber surfaces. In Figure 7, F_max_/areal weight vs. drug loading capacity is shown, whereby the PCL-2 scaffolds prove to be the best performing samples in this study. The dashed line in Figure 7 illustrates the direction of increasing scaffold performance regarding the resistance against suture pullout and drug loading capacity.

## 3. Materials and Methods

### 3.1. Electrospinning

Scaffolds were produced using electrospinning. The well-known technique is extensively described in [22,23,26] and a comprehensive review of electrospinning for tissue engineering can be found in [27]. Briefly, a polymer solution is extruded into an electric field and loaded with charge carriers. As a consequence of repulsive forces, the polymer jet is stretched and collected at a counter electrode, resulting in a random oriented deposition of nanofibers. The experimental setup consists of a glass syringe in which the polymer solution is filled. A stainless 18 gauge steel needle is attached to the syringe and a high voltage source is connected. The syringe is placed in a medical syringe pump to ensure a constant flow rate of the polymer solution. At a specified distance, a grounded plate collector is placed. The collector is wrapped in aluminum foil for easier handling of the scaffolds after the electrospinning process. Thus, rectangular scaffolds with 6 cm in width and 8 cm in height were produced from which later on the individual samples were cut. By varying the polymer concentration or solvent system, different fiber morphologies, especially fiber diameter, can be fabricated. Polymer concentration was altered from 5 g/100 mL to 16 g/100 mL and two different solvent systems were used. With different polymer concentrations as well as different solvent systems, the viscosity of the polymer solution, known to be the main influence on the fiber diameter, can be significantly changed. Larger fiber diameters are observed for increasing solution viscosities [23]. In the present study, polycaprolcatone (PCL, M_w_ = 80 kg/mol, Sigma Aldrich, Saint Louis, MO, USA) nanofibers were produced from the solvent system ethanol / chloroform (ratio 7:3, both Carl Roth GmbH + Co. KG, Karlsruhe, Germany) and formic acid / acetic acid (ratio 7:3, both Carl Roth GmbH + Co. KG, Karlsruhe, Germany). Following preliminary spinning experiments, scaffolds were fabricated with the parameters shown in Table 2.

### 3.2. Scaffold Characterisation

An exact understanding of the scaffolds’ structure is necessary to understand the resulting mechanical properties, resulting drug loading mechanism and release kinetics. Therefore, the scaffolds were examined regarding fiber diameter D, scaffold thickness t, porosity P and areal weight. The latter is a common characteristic from the nonwoven polymer or paper industry, usually denoted in g m^−2^. In the present study, this concept has been adopted for the characterization of nanofibrous scaffolds from electrospinning, since electrospun scaffolds can be classified as nonwovens. Fiber diameters were evaluated using SEM images (CrossBeam, Carl Zeiss Microscopy GmbH, Oberkochen, Germany) and ImageJ software (v1.53o, National Institutes of Health, Bethesda, MD, USA). Therefore, the scaffolds were mounted on SEM sample holders using conductive double sided adhesive carbon tabs and sputter coated with gold using a Q150T Turbo-pumped Sputter Coater (Quorum Technologies Inc., Guelph, ON, Canada). SEM images were taken at a voltage of 2 kV. Scaffolds were produced with different spinning times, resulting in a set of different scaffold thicknesses. For each type of scaffold, the exact dependency of the scaffold thickness and associated areal weight was examined. Therefore, circular discs with a diameter of 10 mm were cut out from the collected sheets. Subsequently, the thickness was measured for every individual sample using a digital contact sensor (GT series, Keyence, Itasca, IL, USA) and weight using a laboratory scale (XA105DU, Mettler Toledo, Giessen, Germany). For the thickness measurement, the scaffolds were sandwiched between a flat base and a circular glass platelet (4.5 mm in diameter). Consequently, the net thickness was measured over a scaffold area of approximately 16 mm^2^. Due to the small sample dimensions, compared to the size of the collector, a homogenous thickness can be presumed for every individual sample. Hence, the areal weight can be calculated with

(4) $$\mathrm{areal}\;\mathrm{weight}=\frac{m}{A}$$

with m the mass and A the area of the sample geometry. For comparing different types of nanofibrous scaffolds, we suggest using areal weight instead of scaffold thickness. Using areal weight, information about the structure of the scaffold are included and thus, a more detailed analysis about the suitability of scaffolds for corneal wound dressing can be conducted. For a specific scaffold, areal weight and scaffold thickness are linearly correlated and scaffold thickness can be calculated from weight measurements, thus a noncontact thickness measurement can be established.

The linear proportional factor between the scaffold thickness and the sample weight for a defined sample geometry is the scaffold density ρ_scaffold_

(5) $$\rho_{\mathrm{scaffold}}=\frac{m_{\mathrm{sample}}}{V_{\mathrm{sample}}}=\frac{\mathrm{areal}\;\mathrm{weight}}{t}$$

or the more commonly used scaffold porosity, describing the percentage of free volume within the scaffold. Scaffold porosity is calculated as

(6) $$P=\left(1-\frac{\rho_{\mathrm{scaffold}}}{\rho_{\mathrm{material}}}\right)\;*\;100\%$$

with ρ_material_ the density of the raw material PCL (ρ_PCL_ = 1.145 kg m^−3^, Sigma Aldrich, Saint Louis, MO, USA). Meanwhile areal weight and thickness are specific values for individual scaffolds, the porosity should be an intrinsic characteristic of each individual sample group.

### 3.3. Suture Retention Test

Corneal wound dressings are usually sewed to the cornea. To quantify the suitability of electrospun nanofibrous scaffolds for the application as wound dressing, suture pullout is of crucial importance. Kueng et al. [24,28] established a suture retention test (SRT) to characterize the ability of biomaterials as corneal wound dressings. Briefly, the SRT describes the maximum force F_max_ necessary for suture pullout. Besides F_max_, the deformation at maximum force d_s_ as well as the deformation at rupture d_r_ is observed. Therefore, a circular piece of 14 mm in diameter is cut out of the scaffold under investigation. The circular sample is then sutured with a medical suture (USB 4–0, SMI AG, St. Vith, Belgium) in a distance of 1 mm to the rim. Both ends of the suture were knotted, the sample clamped in the tensile testing machine (50 N load cell, Zugfestigkeitsprüfmaschine Frank, K. Frank GmbH, Mannheim, Germany) and a bolt placed inside the resulting loop. Using a pre-tension of 0.003 N ensures for reliable data. SRT was performed with a constant deformation rate of 10 mm/min, as suggested by Kueng et al. [24,28], until complete suture pullout. For comparison of different scaffolds, F_max_ is normalized to the areal weight of each sample, thus a normalized force can be defined as

(7) $$F_{\mathrm{norm}}=\frac{F_{\max}}{\mathrm{areal}\;\mathrm{weight}}$$

As benchmark material, data from the SRT of human amniotic membranes (hAM) were taken from Kueng et al. [24], where SRTs were performed on 20 hAMs. Normalized to the thickness of the membranes, an average resistance against suture pullout of 4.0 ± 1.1 N mm^−1^ was measured.

**Figure 8:**
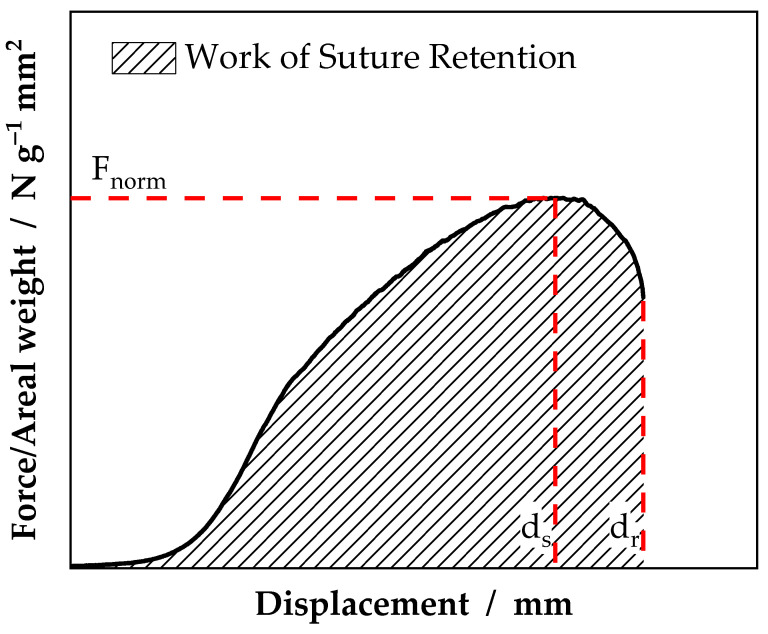
Schematic representation of the experimental data. Form the suture retention test a normalized force-displacement graph can be calculated. The integrated area under the graph defines the necessary work for suture pullout, a characteristic property for a specific scaffold.

In addition to the previously mentioned characteristic SRT parameters, we suggest evaluating the work of suture retention (WoSR) to quantify the necessary work for suture pullout and thus graft failure. The WoSR combines force and displacement, thus accounting for less rigid but flexible biomaterials. In Figure 8, a schematic, normalized force-displacement graph is shown. The WoSR is displayed as integrated area under the graph and the resulting unit can be written as

(8) $$[\mathrm{WoSR}]=\frac{N\;m}{{g\;m}^{-2}}=\frac{J}{{g\;m}^{-2}}=\frac{\mathrm{work}}{\mathrm{areal}\;\mathrm{weight}}$$

A high WoSR defines a sample with increasing resistance against suture pullout. In total, 20 individual samples were tested per sample group.

### 3.4. Layer-by-Layer Coating

Functionalization of PCL scaffolds was realized using layer-by-layer (LbL) coating. With this low-cost and accessible technique, nanostructured layers can be applied to a surface [29,30,31]. Briefly, nanometer thin layers of polyanions and polycations are alternatingly absorbed on a charged substrate. Inserting a desired drug as one of the polyelectrolytes, surfaces can be functionalized as drug carrier.

The LbL-coating and subsequent drug release are based on the pH-dependent solubility of chitosan in aqueous solutions. The primary amine of chitosan has a pKa of approximately 6.4. At low pH, the amino groups are protonated and the chitosan molecules are positively charged, resulting in a high water solubility of chitosan in acid environments and the possibility of applying the LbL process [32,33,34]. With increasing pH, the protonation decreases and thus also the solubility of chitosan in water vanishes. With increasing deprotonation, the electrostatic interactions with the polyanions vanish, thus releasing the previously stable absorbed polyanions. Essentially, the drug release of LbL-coated layers utilizing chitosan as polycation is primarily based on the pKa of chitosan and the associated pH-dependent solubility of chitosan in water. In case of multilayers, the deprotonated chitosan remains as barrier against the burst release of the polyanionic drugs underneath and a decelerated release is realized. The remaining chitosan has been proven to be harmless in ophthalmological environments [35,36,37], and, more generally, in biological environments [38]. In summary, using LbL-coating the drug loading and release kinetics can easily be adjusted with the number of layers, whereby the drug release is initiated with a pH shift from acidic to neutral environments.

In preliminary experiments, the necessity of an ozone treatment (UVCleaner1014, NanoBioAnalytics, Germany) to functionalize the fiber surface was introduced, as the initial PCL surface is uncharged. Using ozone, the C-C bonds were oxidized to carboxyl groups –COO–, thus giving the PCL surface a negative charge [39,40]. A chemical equation is given in the Supplementary Materials in Figure S1. Following the surface activation, alternating layers of polycations and polyanions can be adsorbed on this surface, starting with the positively charged polycations.

In practice, the PCL scaffolds, after the initial surface modification, were immersed in an aqueous solution (50 mM acetate buffer, pH 5.6) of chitosan (Chitopharm S, Cognis, Germany), followed by a washing step with milli-Q-water and then they were immersed in an aqueous solution of heparin or hyaluronic acid (1 g L^−1^, Fluka, Switzerland). For analysis, the heparin was labeled with the fluorescence dye fluorescein and the hyaluronic acid with rhodamine by covalently linking the fluorescent dyes to the polyelectrolyte molecules. Briefly, the heparin or hyaluronic acid was dissolved in water, activated with EDC/NHS and then mixed with amino fluorescein (Sigma Aldrich, Saint Louis, MO, USA) or self-synthesized amino rhodamine (at pH 4.5 and pH 6.5, respectively) and incubated overnight. This was followed by dialysis using a MWCO 15 kDa membrane until no excessive dye was found in the supernatant. The products were freeze dried and the absence of free dye molecules was controlled via thin layer chromatography. Once again, the sheets were washed and a second layer of the polycation can be applied. This way, the desired number of layers was coated.

### 3.5. Drug Release

Following the LbL-coating, drug release studies were performed. Therefore, the pH shift, as shown in Section 3.4, led to the release of heparin and hyaluronic acid, respectively. After the coating, the scaffolds were immersed in phosphate buffered saline (PBS) with a pH of 7.2, triggering the drug release. Using a Fluorimeter (Cary Eclipse, Agilent, Santa Clara, CA, USA) the time-dependent release can be observed. In preliminary experiments, the standard curves for the fluorescence intensity vs. solution concentration were generated (Figures S2 and S3 in Supplementary Materials). Thus, the linear range could be identified and the solution concentration can be evaluated using fluorescence intensity. Therefore, an excitation wavelength of 488 nm and an emission wavelength of 516 nm was set for the hyaluronic acid coated samples and 555 nm and 579 nm for the heparin coated samples, respectively. Measuring the emitted light intensity and normalizing it to the light intensity of the amount of highest possible drug release, the time-dependent percentage amount of released drug can be calculated as

(9) $$\mathrm{drug}\;\mathrm{release}\;(t)=\frac{I(t)}{I_{\max}}\;*\;100\%$$

After two hours, no significant increase in intensity was measured, indicating that the highest possible release of the drug molecules is terminated within this time for the samples in this study. Therefore, $I_{\max}$ was set to be I(t=2 h). The influence of the number of coated layers is demonstrated on fluorescent-labelled heparin-coated samples with one or two layers. Drug release kinetics were characterized with the t_50%_ and t_90%_ values, indicating the time for a drug release of 50% and 90%, respectively.

For the evaluation of the influence of fiber diameter on the drug immobilization properties, fluorescence-labeled hyaluronic acid coated samples were used and the drug storing capacity evaluated using an UV-vis spectrometer (Cary50, Agilent, Santa Clara, CA, USA). Therefore, the absorbance of fluorescent-labelled drug molecules in the supernatant after 2 h stirring in PBS was measured. Similar to the setting for the drug release kinetics, the absorbance was measured at a wavelength of 488 nm. Using the LbL-coating technology, four layers of hyaluronic acid were coated onto the four types of scaffolds. Prior to the coating, the scaffolds were weight. From the final absorbance in the UV-vis spectra, the amount of released drug m_drug_ can be calculated and normalized to the initial scaffold weight m_scaffold_. Thus, drug loading can be defined as

(10) $$\mathrm{drug}\;\mathrm{loading}=\frac{m_{\mathrm{drug}}}{m_{\mathrm{scaffold}}}*100\%$$

## 4. Conclusions

Electrospun scaffolds as artificial substitutes, for example as corneal wound dressings, offer promising opportunities such as adjustable characteristics. Beside mechanical properties, including resistance against suture pullout, a further functionalization of the scaffolds adds beneficial features. Utilizing the scaffolds as drug carrier represents the most obvious method of functionalization. Therefore, in this study scaffolds were coated with either heparin or hyaluronic acid and the influence of decreasing fiber diameter on the resistance against suture pullout and drug loading capacity was investigated. Using the areal weight to normalize the maximum force in the SRT, the PCL-2 scaffolds could be identified as the scaffold with the highest resistance against suture pullout. Remarkably, the PCL-2 scaffolds also exhibited the highest drug loading capacity (Figure 7). Although the specific surface of a scaffold increases with decreasing fiber diameter, a lower limit could be defined, where the functionalization of the scaffold, using the LbL-coating technology, becomes inefficient. In summary, the PCL-2 scaffold, consisting of nanofibers with a mean diameter of 113 nm combines a high resistance against suture pullout as well as high drug loading capacity.

## Figures and Tables

**Table 1:** Fiber diameter, scaffold densities and resulting scaffold porosities of the electrospun nanofibrous scaffolds. PCL-2 deviates from the other scaffolds with a lower scaffold density and thus increased porosity.

Sample	Fiber Diameter (nm)	Scaffold Density (kg m^−3^)	Porosity (%)
PCL-1	35 ± 13	0.18 ± 0.01	84 ± 1
PCL-2	113 ± 22	0.12 ± 0.00	89 ± 0
PCL-3	167 ± 35	0.17 ± 0.01	85 ± 1
PCL-4	549 ± 225	0.17 ± 0.01	85 ± 1

**Table 2:** Electrospinning parameter for the fabrication of nanofibrous scaffolds from two different solvent systems and varying polymer concentrations.

Sample	Concentration (g/100 mL)	Solvent System	High Voltage (kV)	Distance (cm)	Flow Rate (mL/h)
PCL-1	5	formic acid/acetic acid	15	15	0.20
PCL-2	12	formic acid/acetic acid	15	17	0.20
PCL-3	16	formic acid/acetic acid	15	15	0.18
PCL-4	12	ethanol/chloroform	20	22	1.00

## Data Availability

The data presented in this study are available on reasonable request from the corresponding author.

## Supplementary Materials

The following supporting information can be downloaded at: https://www.mdpi.com/article/10.3390/ijms23052765/s1.
