# Supplementary material for: Electrospun PCL Scaffolds as Drug Carrier for Corneal Wound Dressing Using Layer-by-Layer Coating of Hyaluronic Acid and Heparin
Source: Int J Mol Sci. 2022 Mar 2;23(5):2765. doi: 10.3390/ijms23052765 (PMC8910869; doi:10.3390/ijms23052765)
Supplement: Supplementary file 1 [file ijms-23-02765-s001.zip › ijms-1610003-supplementary.pdf]

International Journal of
Molecular Sciences

MDPI

Supplementary Materials

Electrospun PCL Scaffolds as Drug Carrier for Corneal Wound Dressing Using Layer-by-Layer Coating of Hyaluronic Acid and Heparin

Marcus Himmler [1,2]*, Dirk W. Schubert [2],Lars Dähne [3], Gabriella Egri [3] and Thomas A. Fuchsluger [1,*]

1 Department of Ophthalmology, University Medical Center Rostock, Doberaner Straße 140, 18057 Rostock, Germany
2 Institute of Polymer Materials, Friedrich-Alexander University Erlangen-Nuremberg, Martenstraße 7, 91058 Erlangen, Germany; dirk.schubert@fau.de (D.W.S.)
3 Surflay Nanotec GmbH, Max-Planck-Str. 3, 12489 Berlin, Germany; l.daehne@surflay.com (L.D.); g.egri@surflay.com (G.E.)
* Correspondence: marcus.himmler@fau.de (M.H.); thomas.fuchsluger@med.uni-rostock.de (T.A.F.)

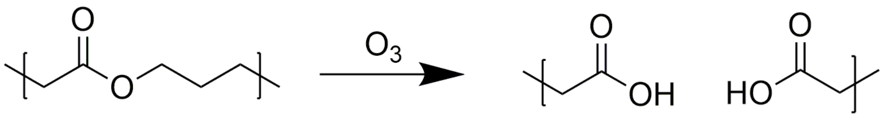

Figure S1. Chemical equation for the generation of carboxyl groups on the surface of the PCL nanofibers.

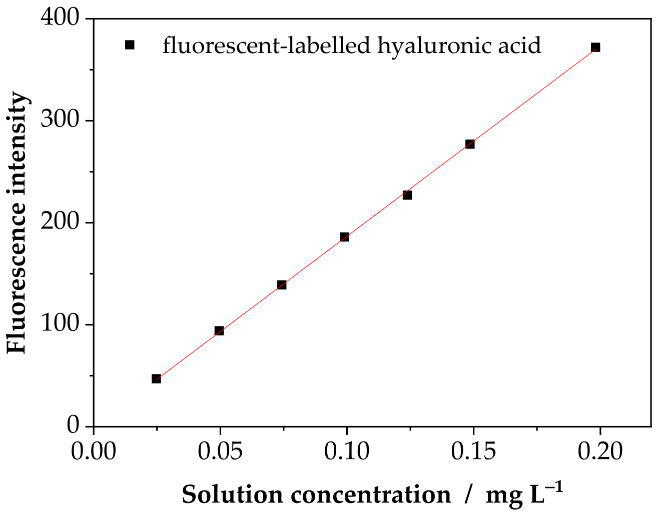

Figure S2. Standard curve for the fluorescence intensity versus solution concentration for fluorescent-labelled hyaluronic acid.

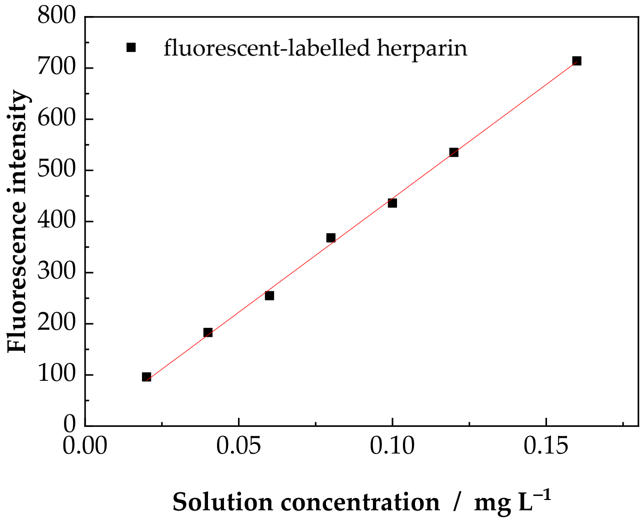

Figure S3. Standard curve for the fluorescence intensity versus solution concentration for fluorescent-labelled heparin.

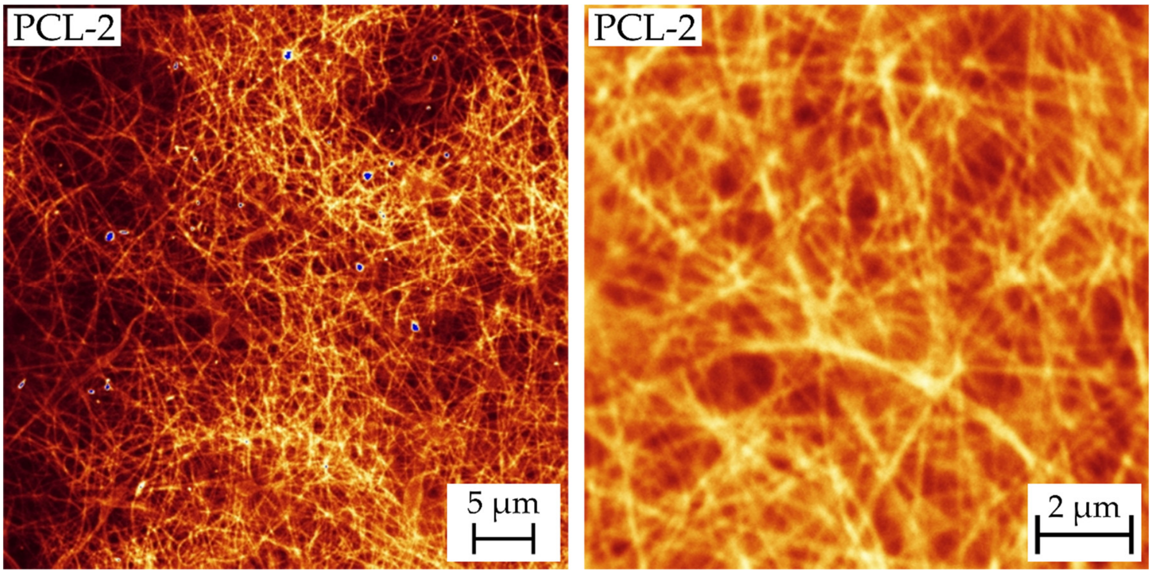

Figure S4. Exemplary Confocal Laser Sscanning Microscopy (CLSM) image of PCL-2 scaffolds LbL-coated with fluorescently labeled heparin. The nanofibrous structure remains intact after LbL coating and can be visualized by CLSM.
